# Resting state brain activity and association with transfer of cognitive training gains

**DOI:** 10.1016/j.ynirp.2026.100389

**Published:** 2026-07-24

**Authors:** Sofia Faraza, Martin Dyrba, Dominik Wolf, Florian U. Fischer, Kristel Knaepen, David Riedel, Bianca Kollmann, Harald Binder, Andreas Mierau, Andreas Fellgiebel, Oliver Tüscher, Stefan Teipel, the German AgeGain Study Group

**Affiliations:** a Department of Psychosomatic Medicine and Psychotherapy, Rostock University Medical Center Rostock, Germany; b University Hospital of Child and Adolescent Psychiatry, University of Bern, Switzerland; c Deutsches Zentrum für Neurodegenerative Erkrankungen (DZNE), Rostock, Germany; d Department of Psychiatry and Psychotherapy, University Medical Center Mainz, Germany; e Department of Psychiatry, Psychotherapy and Psychosomatic Medicine, University Medical Center Halle, Halle (Saale), Germany; f Institute of Movement and Neurosciences, German Sport University Cologne, Cologne, Germany; g Leibnitz Institute for Resilience Research (LIR), Mainz, Germany; h Department of Neuropsychology and Psychological Resilience Research, Central Institute of Mental Health (ZI), Mannheim, Germany; i Institute of Medical Biometry and Statistics (IMBI), Faculty of Medicine and Medical Center, University of Freiburg, Freiburg, Germany; j Department of Exercise and Sport Science, LUNEX International University of Health, Exercise and Sports, Differdange, Luxembourg; k Clinic for Psychiatry, Psychosomatics and Psychotherapy, AGAPLESION Elisabethenstift, Darmstadt, Germany; l German Center for Mental Health (DZPG), Site Halle-Jena-Magdeburg, Halle (Saale), Germany; m Institute of Molecular Biology (IMB) Mainz gGmbH, Mainz, Germany

**Keywords:** Cognitive training, Transfer of gains, Cognitive performance, Resting state functional activity, Healthy aging

## Abstract

**Background:**

Normal aging is accompanied by cognitive decline, structural and functional brain changes. Cognitive training is a potentially effective intervention for cognitive improvement. Transfer of training gains to untrained tasks is the ultimate goal of cognitive training. However, the neural mechanisms underlying successful transfer remain underinvestigated.

**Objective:**

To examine the predictive role of resting-state functional connectivity in the transfer of training gains.

**Methods:**

We analyzed resting-state fMRI and cognitive data of 181 healthy older adults (mean age: 68 years) who underwent a 4-week cognitive training at three study sites. The control group consisted of 54 older adults. Participants underwent neuropsychological assessments before and directly after the training, as well as 12 weeks after. We used aggregate scores representing working memory, memory and executive functions to assess transfer effects. Baseline resting-state fMRI was used to investigate functional connectivity. We used a seed-based and an independent component analysis approach to examine brain network activity.

**Results:**

The majority of our participants transferred cognitive training gains successfully over a three-month period. Baseline resting-state functional connectivity within the default mode network and the central executive network did not predict transfer of training gains.

**Conclusions:**

Baseline resting-state functional connectivity of large-scale networks does not appear to predict who will benefit from cognitive training in healthy older adults. These findings contribute to a better understanding of the functional brain mechanisms underlying transfer of training gains and highlight the need for larger, multi-modal neuroimaging studies to identify reliable neural predictors of cognitive training outcomes.

## Highlights

• Near transfer of cognitive training gains is possible in healthy aging.
• Functional connectivity of the DMN and CEN did not serve as predictors of successful transfer of cognitive training gains.
• A high baseline neuropsychological performance decreases the likelihood of transfer.

## 1 Introduction

Structural and functional changes of the brain during normal aging lead to reduction of cognitive functions, such as executive function, working memory, processing speed and cognitive plasticity (Sánchez-Izquierdo and Fernández-Ballesteros, 2021).

Functional neuroimaging data provide important insight into brain functional changes associated with aging (Grady, 2012). Spontaneous fluctuations of blood oxygenation level-dependent (BOLD) signal, as measured by functional magnetic resonance imaging (fMRI), has revealed a number of functional networks, such as the default mode network (DMN) and the central executive network (CEN), which are involved in cognitive processes (Fox et al., 2005; Lee et al., 2013; Rosazza and Minati, 2011). The DMN is a widely investigated network and important in aging, because it is vulnerable to early stages of Alzheimer's disease (AD) pathology (Sheline et al., 2010) and has been associated with memory functions, mind-wandering and self-monitoring (Rosazza and Minati, 2011). It includes regions, such as posterior cingulate cortex, lateral parietal cortex and medial prefrontal cortex (Rosazza and Minati, 2011). The CEN is a wide network, often also referred to as fronto-parietal network (FPN), which includes frontal and parietal regions and has been linked to higher cognitive processes, such as working memory and cognitive control (Daigle et al., 2022; Rosazza and Minati, 2011).

Cross-sectional studies indicated decreased DMN activity in aging (Damoiseaux, 2017; Dennis and Thompson, 2014; Ferreira and Busatto, 2013; Grady et al., 2016; Jockwitz and Caspers, 2021; Zonneveld et al., 2019). Moreover, some studies found age-related decreases in higher order networks, such as the CEN (Littow et al., 2010), and others found increased activation in the CEN at rest (N. Filippini et al., 2012). Data from longitudinal functional imaging studies provided mixed findings. For instance, they demonstrated functional changes on DMN and CEN connectivity over a period of four years of healthy aging (Ng et al., 2016). In contrast, other longitudinal studies showed a stable average DMN connectivity over a 4 year (Oschmann and Gawryluk, 2020) or even 7 year period (Malagurski et al., 2022) in healthy older adults. Additionally, they demonstrated that some DMN components, such as posterior cingulate cortex (PCC) and parietal cortex showed decreased functional connectivity (FC), and in contrast prefrontal regions showed increased FC over time (Malagurski et al., 2022). These findings indicate a complex pattern of functional changes in DMN during aging. Moreover, they support previous evidence of, possibly compensatory high resting state FC in prefrontal regions in healthy aging (Grady et al., 2016; H.-J. Li et al., 2015). These age-related changes in DMN and CEN connectivity provide the neural context for examining whether cognitive training can modulate functional network organisation, and whether individual differences in baseline FC predict who will benefit most from training.

The Scaffolding Theory of Aging and Cognition (STAC- revised model) suggests that the increased functional frontal activation in aging is evidence of an adaptive brain (Park and Reuter-Lorenz, 2009; Reuter-Lorenz and Park, 2014). Scaffolding is a protective process that enables the recruitment of alternative brain regions in order to compensate for age-related decline and it is strengthened by life-style factors and interventions, such as cognitive training (Reuter-Lorenz and Park, 2014).

Cognitive training is an important intervention in aging, given its perspective to enhance neural plasticity. Recent meta-analyses provide evidence for the effectiveness of cognitive training in healthy older adults, not only for general cognitive function, but also for specific cognitive skills such as memory, attention, executive function and visuo-spatial ability, with moderate to small effect sizes (Chiu et al., 2017; Z. Li et al., 2024; Sanjuán et al., 2020; Yun and Ryu, 2022). Systematic reviews (Gavelin et al., 2020; Yi et al., 2024) demonstrated that combined cognitive and physical interventions can be effective in promoting physical and cognitive health. Cognitive training has also been a recommendation in clinical practice guidelines for patients with mild cognitive impairment (Petersen et al., 2018).

Intervention studies emphasize enhancing the performance of trained skills. However, the ultimate goal of cognitive training is to generalize those skills to other cognitive outcomes and aspects of daily life, namely transfer. There is a common distinction between the generalization of trained skills across similar cognitive domains (*near* transfer) and across domains that are not closely related (*far* transfer) (Barnett and Ceci, 2002). A second-order meta-analysis by Sala et al. (2019) showed that near transfer frequently occurs, though younger populations tend to benefit more from training interventions than older ones. However, they did not find strong evidence of far transfer, regardless of the age group. Another meta-analysis by Gobet and Sala (2023) supports the lack of evidence for far transfer, regardless of the type of training and they recommend future studies to focus on near transfer. Similarly, a systematic meta-review by Velloso et al. (2025) supported the evidence of near transfer and showed that most studies have focused mostly on short-term rather than long-term transfer effects. Moreover, previous meta-analyses have demonstrated the possibility of near transfer in aging (G. Li et al., 2025; Masurovsky, 2020). There are some important factors that might moderate transfer, such as age, education, baseline performance of participants, training dose and length (Teixeira-Santos et al., 2019; Zinke et al., 2014). It should be noted that the heterogeneity in intervention design, methodology, and measurement tools, may lead to discrepant results (Mendes et al., 2022).

A comprehensive understanding of transfer mechanisms could contribute to the design of effective interventions. A review by Baykara et al. (2021) highlighted the importance of the investigation of baseline brain mechanisms for prediction of transfer and maintenance of training gains. A growing body of neuroimaging research has demonstrated that cognitive training leads to changes in resting-state FC, particularly within the DMN, CEN, and salience networks (Beaumier et al., 2025; Cao et al., 2016; Chapman et al., 2015; Dimitriadis et al., 2024; Yan et al., 2025). For instance, Kang et al. (2024) revealed an association between high FC on hippocampal and fusi-form occipital regions and memory transfer in patients with mild cognitive impairment. In a different study, Kang et al. (2021) demonstrated an association between increased frontal-occipital FC and improved visuospatial performance following virtual reality cognitive training in patients in a predementia state. These post-training connectivity changes confirm that resting-state fMRI is sensitive to training-induced neuroplasticity. However, the question of whether baseline resting-state FC can predict who will benefit from training has received less attention (Anthony et al., 2024; Gallen et al., 2016 investigated the predictive role of brain network modularity and demonstrated that healthy older adults with higher cortex sub-networks at baseline, such as the default mode and fronto-parietal, exhibited greater cognitive training gains. Nikolaidis, 2016 demonstrated the predictive role of DMN and FPN for training gains and transfer in working memory in young adults. Our previous study (Faraza et al., 2021) showed that high resting-state FC of the dorsolateral prefrontal cortex (dlPFC) predicted transfer of gains in the visual working memory domain.

Considering the sparse evidence in the literature, we aimed to investigate the predictive role of FC of brain networks during resting state in the transfer of cognitive training gains. First, we aimed to determine whether multimodal computerized cognitive training of healthy older adults could have near transfer effects. Based on previous studies, we hypothesized that near transfer would occur and training gains would be maintained over a 3-month period (Dahlin et al., 2008a; Velloso et al., 2025). Second, we sought to investigate if a higher FC of the DMN and CEN would be associated with the transfer of cognitive training gains directly after the training and three months later in healthy older adults. Our previous study (Faraza et al., 2021) showed the predictive role of high FC of dlPFC in the transfer of training gains. In light of our recent findings, we extended our analyses to examine the FC of large-scale networks. We hypothesized that high FC of the DMN and CEN would be associated with transfer of cognitive training gains. In addition, we examined demographic characteristics as potential predictors for training gains based on previous studies (Bruno et al., 2024; Roheger et al., 2020).

## 2 Materials and methods

### 2.1 Selection of participants and procedures

**Fig. 1:**
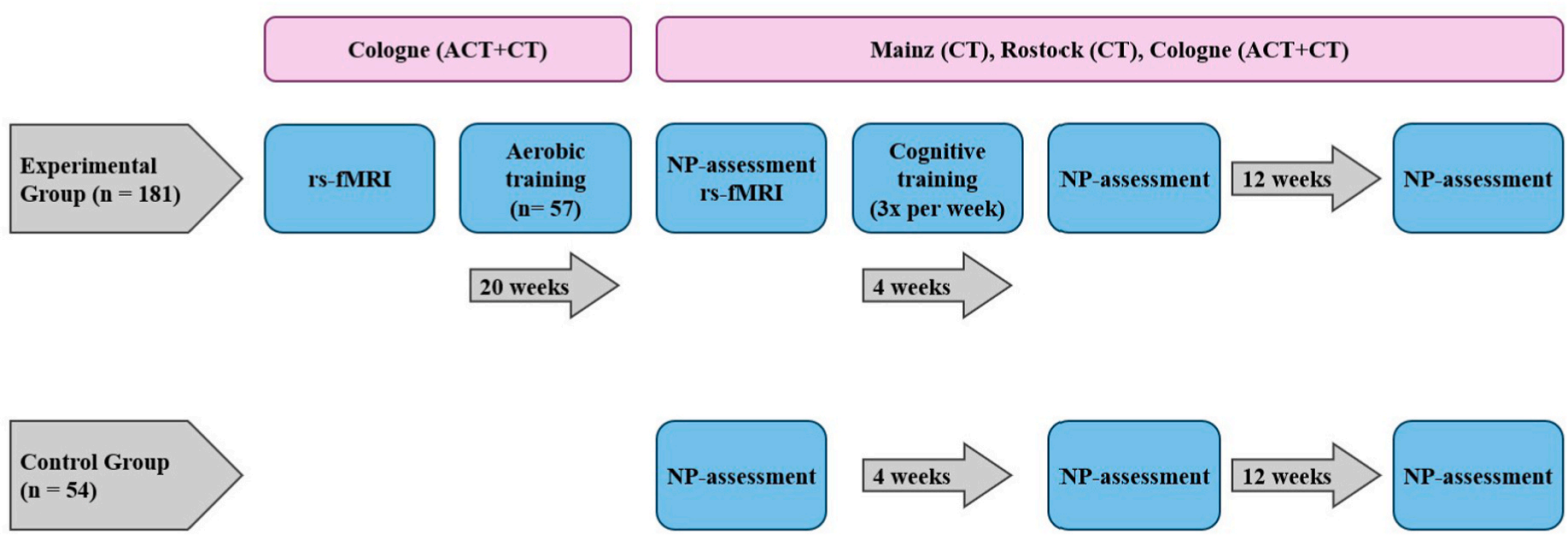
Study design.

Data used in this article were obtained from the AgeGain study (Wolf et al., 2018), a longitudinal, interventional, multi-center, multi-modal imaging trial (German Clinical Trials Register, ID: DRKS00013077). The AgeGain study aimed to reproduce successful transfer of training gains and investigate the underlying brain mechanisms. A more detailed description of the study and information about inclusion and exclusion criteria, can be found in the study protocol (Wolf et al., 2018). Participants were recruited through announcements in local newspaper and flyers in Germany and they enrolled by the University Medical Centers in Mainz and Rostock, and the German Sport University Cologne. Our sample consisted of 235 healthy older adults (age range 60-88 years). The experimental group consisted of 181 participants who underwent a 4-week cognitive training (CT). A subgroup of 57 participants had additional aerobic and coordination training (ACT + CT) for 20 weeks, before the cognitive training and the baseline neuropsychological assessment. Baseline resting-state fMRI scans and three neuropsychological assessments were obtained from the experimental group. The control group (CG) consisted of 54 participants who underwent three neuropsychological assessments and did not undergo cognitive training or engage in other activities (passive, no-contact control group). The first neuropsychological assessment occurred before the training, the second directly after and the third assessment 12 weeks after the training (Fig. 1).

### 2.2 Pre-registration and deviations from the study protocol

The primary endpoint of the AgeGain trial was the prediction of cognitive training gain transfer using corpus callosum integrity and bihemispheric processing, which has been reported by Fischer et al. (2025) as the primary behavioral report of the trial. Faraza et al. (2021) reported results from an interim data freeze. The present paper reports secondary neuroimaging analyses focused on resting-state FC as a predictor of cognitive training transfer. Resting-state fMRI connectivity is explicitly listed as a secondary outcome in the registered study protocol (Wolf et al., 2018, Table 1). The present analyses were conducted within the framework of the approved protocol by members of the AgeGain study group.

**Table 1:** Demographic characteristics of total sample and across 3 centers.

	Full sample	Mainz	Rostock	Cologne
	*n*	*n*	*n*	*n*
Participants	235	74	71	90
Groups				
*CT*	124	56	54	14
*Control*	54	18	17	19
*CT + ACT*	57			57
Sex^a^				
*Female*	117	37	43	37
*Male*	118	37	28	53
Age(SD)^a^	68.4(5.6)	69.5(5.5)	68.2(6.0)	67.7 (5.1)
Years of education(SD)^a^	15.7(2.7)	15.6(2.9)	15.5(2.2)	16(2.8)
Verbal IQ(SD)^a^	111.6(9.0)	112.9(10.2)	108.7(7.5)	112.8(8.6)

*Note*. *CT*= Cognitive Training, *CT + ACT =* Cognitive Training + Aerobic Coordination Training; ^a^ Sites did not differ in demographics.

The following elements were pre-specified in the study protocol: (1) resting-state fMRI of the DMN as a secondary neuroimaging outcome, (2) binary transfer classification (improvement exceeding the control group mean change), (3) logistic regression as the primary statistical approach for the secondary FC analyses. The following elements were not pre-specified in the study protocol: (1) the seed-based FC methodology and the specific seed regions of interest, which were selected based on prior literature, (2) the CEN as a target network, because the protocol specified DMN only, (3) ICA-based network analyses (exploratory complement), (4) continuous linear regression analyses added as co-primary,(5) CT-only sensitivity analyses. The following deviations from the study protocol occurred: (1) transfer outcome redefined from individual task scores (LPS-4 only) to aggregate domain scores across executive function, memory, and working memory, (2) continuous analyses elevated to co-primary status alongside the pre-registered binary classification.

### 2.3 Randomization and blinding

The assignment to the experimental and control groups was based on randomization and stratified by the trial coordinator at the University Medical Center Mainz trial site. The study code was centrally managed by the coordinator site. Written informed consent was obtained by all participants and the study protocol was approved by the respective local ethics commission. Participants were aware of their group assignment as the nature of the cognitive training intervention precluded blinding of participants to condition. Cognitive training was delivered by experimenters at each study site. Neuropsychological assessments were conducted by a separate team of neuropsychologists who were not involved in training delivery, reducing the risk of performance bias during training. The neuropsychologists were aware of participants' group assignment. However, the neuropsychological assessments were administered using standardised tests with objective scoring procedures, minimising the risk of bias in outcome measurement despite neuropsychologists not being formally blinded to group assignment. Resting-state fMRI data were acquired exclusively from experimental participants (CT and ACT + CT groups). The control group did not undergo baseline fMRI acquisition. The neuroimaging analysis was conducted using fully automated pipelines, as described in section2.8, minimising the bias of the researcher.

### 2.4 Definition of primary endpoint

The primary endpoint of this study was defined as dichotomous outcome training gain transfer (Yes/No). In the original study protocol (Wolf et al., 2018) successful transfer was defined as: 1) performance improvement in an untrained fluid intelligence task (Leistungspruefsystem subtest 4- logical reasoning (LPS-4) (Horn, 1983) from the pre-training to post-training phase, 2) maintenance of performance improvement from post-training to follow-up phase, and 3) performance improvement on logical reasoning training task from the second to the last training session. Performance improvement has to be greater than in the untrained control group (Wolf et al., 2018). After an internal check of the data freeze, we found that most of the participants did not have successful transfer on LPS-4. Moreover, we observed that the post-training cognitive performance on LPS-4 was lower than the baseline and follow-up performance. Training performance on the logical reasoning training task was improved from the second to the last training session. Based on these observations and findings, the AgeGain study committee decided to use a broader definition of transfer and include more cognitive transfer tasks. Successful transfer was re-defined as: 1) performance improvement on untrained tests from pre-training to post-training phase (short-term transfer), 2) maintenance of improvement on untrained tests from post-training to follow-up phase (maintenance of training gains), and 3) improvement on untrained tests from pre-training to follow-up phase (long-term transfer). The improvement on the neuropsychological assessment had to be greater than in the control group, in order to account for retest effects. The transfer tasks will be described in section 2.5.

### 2.5 Cognitive training

The training was conducted at the study sites under the supervision of an experimenter. Each participant was seated in front of a laptop equipped with headphones and training software. Participants of CT and ACT + CT groups underwent computerized cognitive training which consisted of 12 sessions (1 h/session) for 4 weeks, with 3 sessions per week. “Training and Testing Tool” (TATOOL, www.tatool.ch) was used to train working memory functions (Bastian et al., 2013). We included the subtests “Tower of Fame” and “Complex Span” from TATOOL, which were adaptive and the difficulty level was increasing based on the performance of each participant. For attentional functions, we used the “Test for Attentional Performance” battery (TAP - Test of Attentional Performance, 2024; Version 2.1.3., Zimmermann and Fimm, PsyTest, 2024). From this battery we included the subtests of “Divided attention” and “Altertness”. Executive functions, memory and processing speed were trained by “The Cognitive Training Package” (Cogpack, https://www.cogpack.de/D/frames.htm, Version 7.6., Marker K., 2001). The tasks we used were Comparison, Searching, Logic, Anagrams, Complete a Logical Block and Remembering. The tasks from TAP and Cogpack had to be repeated over and over again throughout the training sessions. The tasks from TATOOL were adaptive. More details about the tasks can be found on the Supplementary File 1.

### 2.6 Neuropsychological assessment and aggregate measures

Participants completed a battery of neuropsychological tests. For the current analyses, we selected tests representing memory, working memory and executive functions from the full neuropsychological battery (Wolf et al., 2018). More specifically, memory was measured by the delayed recall and recognition tasks of Verbal Learning Memory Test (VLMT), a German version of the auditory verbal learning test (Helmstaedter et al.) and by the immediate and delayed recall tasks of the Visual Paired Associations, a subtest of the Wechsler- Memory Scale-revised (WMS-R) (Wechsler, 1987). Working memory was measured by the subtests digit span and block span (forward and backward) of the Wechsler- Memory Scale-revised (WMS-R) (Wechsler, 1987). Executive functions were assessed by Trail Making Test B (R. Reitan, 1992), Tower of London (Shallice, 1982), Leistungspruefsystem (LPS) (comparable to Raven Matrices): subtest 4 (LPS-4: logical reasoning) and 8 (LPS-8: visuospatial perception) (Horn, 1983). Including the abovementioned neuropsychological tests we built three cognitive domains: Memory, Working memory and Executive functions. This was realized by standardizing all raw variables as T-scores based on distribution parameters of the baseline neuropsychological assessment and then calculating the mean score of each time point of the assessment. For visualisation and comparability across domains, aggregate scores were z-standardised across all participants prior to plotting.

### 2.7 Imaging data acquisition and preprocessing

The MRI scans were acquired from three 3T Siemens scanners (1 Verio, 1 Prisma, 1 Trio) using identical acquisition parameters and harmonized instructions. T1-weighted anatomical images were acquired using a Magnetization Prepared Rapid Gradient Echo (MPRAGE) sequence with the following parameters: sagittal slices = 176, scan time = 4.18 min, repetition time (TR) = 1900 ms, echo time (TE) = 2.45, flip angle = 9°, field of view (FOV) = 250 mm, voxel volumes = 1.0 × 1.0 × 1.0 mm. Resting-state functional MRI (rs-fMRI) scans were acquired with the following parameters: scan time = 11.02 min, transversal slices = 60, slice thickness = 2.5 mm, TR = 1000 ms, TE = 30.6 ms, flip angle = 56°, FOV = 210 mm. Participants were instructed to keep their eyes closed and remain awake.

The resting-state fMRI scans were processed using the Data Processing Assistant for Resting-State fMRI (DPARSFA, Version 4.3, Chao-Gan and Yu-Feng, 2010), implemented in MATLAB (MATLAB, Version, 2020a; The MathWorks) in conjuction with the statistical parametric mapping software (SPM12, Welcome Trust Center for Neuroimaging, https://www.fil.ion.ucl.ac.uk/spm/). We removed the first 10 vol of each fMRI scan and then we applied series of steps, such as slice timing correction and realignment to remove timing differences and head movement artifacts. The T1-weighted MPRAGE scans were coregistered to the mean fMRI images, segmented into white matter (WM), gray matter (GM) and cerebrospinal fluid (CSF), and spatially normalized to Montreal Neurological Institute (MNI) space using the Diffeomorphic Anatomical Registration Through Exponentiated Lie Algebra (DARTEL) algorithm (Ashburner, 2007). To reduce the influence of noise, we regressed out linear trend, 24 motion parameters (6 head motion parameters, 6 head motion parameters one time point before, and the 12 corresponding squared items) (Friston et al., 1996), white matter, cerebrospinal fluid and global signal as nuisance regressors. Global signal regression (GSR) was selected as the primary denoising approach because it effectively reduces global noise across voxels and it improves anatomical specifity of the connectivity maps, which is advantageous in seed-based connectivity analyses where sensitivity to regional neural signal is the primary goal (Fox et al., 2009). Moreover, it increases the behavioral correlations with connectivity patterns (J. Li et al., 2019). In addition, a recent study by Golestani and Chen (2024) compared denoising methods and showed that GSR and ICA AROMA were more effective in removing physiological noise (cardiac and respiratory BOLD-signal power) followed by aCompCor. The functional images were filtered with a band-pass filter between 0.01 and 0.1 Hz. The resulting nonlinear deformation fields were then applied to the frequency-filtered fMRI data to transform them to MNI reference space. Finally, fMRI scans were resampled to 3 mm × 3 mm × 3 mm voxel size and smoothed with a 6 mm full-width-at-half-maximum (FWHM) Gaussian kernel. All scans were controlled for excessive head motion (>3.0 mm) and no participants were excluded. Mean framewise displacement (FD) (Jenkinson et al., 2002) was 0.194 mm (SD = 0.087, range: 0.056–0.812 mm), indicating low levels of head motion overall.

### 2.8 Data analysis

#### 2.8.1 Operationalisation of transfer of training gains

Transfer of training gains was examined using two co-primary approaches. First, as the primary statistical test, continuous z-standardised cognitive change scores were used as outcomes in linear regression models. Second, to ensure comparability and consistency with the study design (Wolf et al., 2018), a binary classification of transferers was applied as described below. As mentioned in section 2.4., successful transfer was redefined relative to the study protocol to give a broader definition and include more cognitive tasks. Based on this redefinition we classified our participants into groups that had or had not successful short-term transfer (ST+, ST-), maintenance of training gains (TM+, TM-) and long-term transfer (LT+, LT-). We measured the difference in cognitive scores between two assessments of the experimental group, and if this difference was greater than the mean score difference of the control group, then the participant was classified as a successful transferer (1) or, in the opposite case, as a non-successful transferer (0). Specifically, we performed the following calculations: 1) for short-term transfer: Δ = Post-training assessment score – Pre-training assessment score > mean difference score of control group, 2) for maintenance of training gains: Δ = Follow-up assessment score – Post-training assessment score > mean difference score of control group and 3) for long-term transfer: Δ = Follow-up assessment score – Pre-training assessment score > mean difference score of control group. We examined the differences in cognitive performance based on aggregate scores of three cognitive domains. In the present study we investigated near transfer effects by examining performance on untrained cognitive tasks that share the same cognitive domains, as the trained tasks.

#### 2.8.2 Baseline performance of CT and ACT + CT groups and transfer effects

First, we wanted to examine whether there was a difference in cognitive performance between the CT and ACT + CT groups to assess whether the ACT + CT group had a cognitive advantage due to the additional aerobic training. Therefore, we compared the distribution scores of the baseline cognitive performance of the two groups in executive functions, memory and working memory domains using two-sample Kolmogorov-Smirnov tests. In addition, we examined near transfer effects of cognitive training. We used R (version 4.5.0; R Core Team, 2025) and the *lme4* and *lmerTest* packages (Bates et al., 2015) to perform mixed effects analyses to assess differences in performance between CT, ACT + CT and CG groups across three time points. We fitted a fixed effect model using the aggregate scores of the cognitive domains as dependent variables. Groups, time of the three assessments and their interaction (group by time) were entered as fixed effects and intercepts for subjects as random effects. Sex, age, education and site were included as covariates to control for demographic differences and inter-site variability. Effect sizes were calculated as partial eta squared (η^2^p) using the *effectsize* package (Ben-Shachar et al., 2020). Aggregate scores were z-standardised prior to analysis to allow direct comparison of effect sizes across cognitive domains.

### 2.9 Resting-state fMRI data analyses

To examine the association between baseline resting-state FC and cognitive training transfer, two neuroimaging approaches were conducted. First, seed-based FC analyses were conducted as the pre-registered primary neuroimaging approach, examining both the continuous relationship between baseline FC and cognitive change scores and the pre-registered binary transfer classification. Second, independent component analyses were conducted as an exploratory complement, providing a data-driven, network-level examination of FC differences between transferers and non-transferers.

#### 2.9.1 Resting-state global seed-based connectivity

We first excluded 6 participants from the total sample due to missing fMRI scans. Thus, the sample for the resting-state FC analyses was 175. It should be noted that we analyzed the resting-state fMRI scans, which were obtained at baseline, before any kind of training was performed. We conducted seed-based connectivity analysis using *a priori* regions of interest based on previous literature. More specifically, we used seeds of posterior cingulate cortex (PCC) (MNI coordinates: 0,-51,15) (Weiler et al., 2014), medial prefrontal cortex (mPFC) (−2,58,-6) and lateral parietal cortex (lPC) (46,-60,32) (M.-T. Li et al., 2023) as most representative regions of DMN. For CEN, we used right (45,18,48) and left (−45,18,48) dorsolateral prefrontal cortices (dlPFC) (Weiler et al., 2014) and anterior cingulate cortex (ACC) (2,36,22) (M.-T. Li et al., 2023) as representative regions of CEN.

We obtained seed-based connectivity (SBC) maps representing the FC of each seed region of interest. This resulted in six SBC maps. Subsequently, we performed global FC (gFC) analyses to measure the strength of FC of the regions of interest. Our aim was to examine the gFC of each region separately. To perform these analyses we used a script from Franzmeier et al. (2017) which included the following steps: 1) we obtained a correlation of each voxel's resting-state time series with those of the seed-regions, 2) the correlations were transformed to Fisher Z values, thresholded at z > 0.3 and averaged to produce a global FC value. Only positive correlations were included because they reflect higher connectivity strength (Cole et al., 2012).

As a primary analysis of the association between baseline resting-state FC and transfer of gains, we conducted linear regression analyses predicting continuous cognitive change scores from baseline gFC. Continuous analyses were prioritised as the primary approach because they are more sensitive and avoid the conceptual limitation of binary classification thresholds. For each cognitive domain and transfer type, we computed a continuous change score by subtracting the baseline aggregate score from the post-training or follow-up aggregate score. For each of the six seed regions of interest, a separate linear regression model was fitted with the continuous change score as the dependent variable, and baseline gFC, age, sex, education, site and baseline performance as predictors. All outcome variables were z-standardized prior to analysis. False discovery rate (FDR) correction was applied across all p-values using the Benjamini-Hochberg procedure.

In addition, to ensure consistency with the study protocol (Wolf et al., 2018), we conducted secondary logistic regression analyses using the binary transfer classification as the outcome. Logistic regression was performed using R (version 4.5.0; R Core Team, 2025) to ascertain the likelihood of having successful short-term, maintenance and long-term transfer of training gains with higher gFC, using gFC of each region of interest as predictor. Sex, age, education, site and baseline performance were included in each model as covariates. We z-transformed the variables and built separate models for each region of interest. In addition, to ensure that the inclusion of the ACT + CT group did not influence the results, we performed sensitivity analysis only with the CT group for both the linear and logistic regression models for more transparent results.

#### 2.9.2 Independent component analysis

The extraction of resting-state FC maps was carried out using Independent Component Analysis (ICA) (Beckmann et al., 2009) implemented in the MELODIC (Multivariate Exploratory Linear Decomposition into Independent Components) toolbox of FSL (FMRIB Software Library, Version 6.0, Oxford, UK, https://fsl.fmrib.ox.ac.uk/fsl/docs/index.html). The resulting 30 ICA maps were evaluated based on their spatial patterns to identify the resting-state networks. We used FSL's dual regression, which generated subject-specific versions of the ICA maps and associated time-series to derive subject-level resting-state FC z-maps. More specifically, the group-average set of spatial maps was regressed into each subject's 4D dataset. This resulted in a set of subject-specific time-series. Next, these time-series were regressed into the same 4D dataset, resulting in a set of subject-specific spatial maps (Beckmann et al., 2009; Filippini et al., 2009; Nickerson et al., 2017). To define the FC areas which actually belong to the corresponding resting-state networks, we obtained network-specific masks from the ICA maps of the whole sample, thresholded at p < .05 TFCE (Threshold-Free Cluster Enhancement). Our selected ICA-derived networks corresponded to DMN and CEN components (Rosazza and Minati, 2011). For the selection of the networks we used templates of functional regions of brain networks, as previously reported in Shirer et al. (2012), which were matched to our extracted components. We then regressed each contrast of interest on the respective resting-state functional network. All the contrasts (e.g. transfer, sex, age, education, site, baseline performance) were included in each model built for each network. We tested voxel-wise for statistically significant differences across subjects using FSL's randomize permutation-testing tool (Winkler et al., 2014).The resulting *t*-test statistic images (stat) were thresholded at p < .01, uncorrected for each contrast.

## 3 Results

### 3.1 Demographics

Demographic characteristics of total sample and separately by recruiting center can be found in Table 1. Demographic characteristics of the training and control group can be found in Table 2. The results demonstrated no significant differences across sites and groups.

**Table 2:** Demographic characteristics across groups.

	Training group	Control group
	*n*	*n*
Participants	181	54
Sex		
*Female*	90	26
*Male*	91	28
Age(SD)^a^	68.3(5.6)	68.8(5.7)
Years of education(SD)^a^	15.8(2.7)	15.5(2.8)
Verbal IQ(SD)^a^	111.1(8.9)	113.2(9.3)

*Note*. ^a^ Groups did not differ in demographics.

### 3.2 Transfer/No transfer

**Fig. 2:**
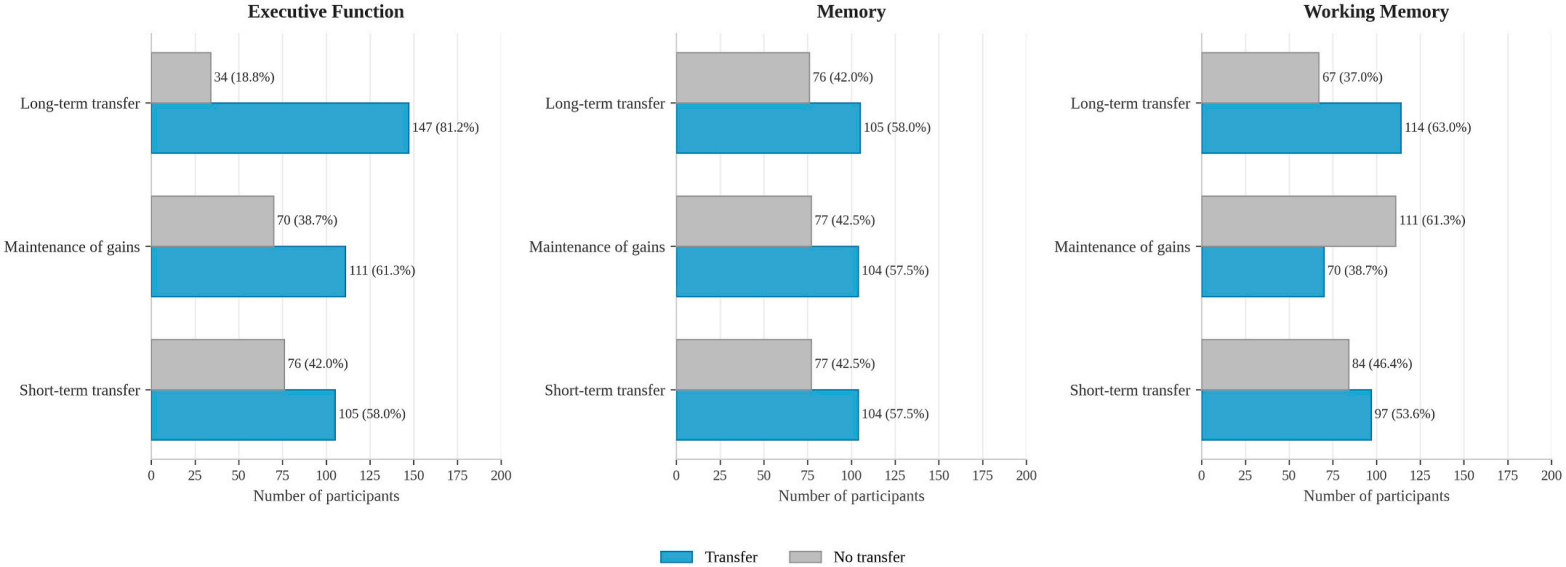
Transferers and no transferers of the sample.

The number of participants with and without transfer on three cognitive domains is listed in Fig. 2. Our results showed that 58% of all of our participants had short-term transfer over 4 weeks, 61,3% maintained gains over 3 months and 81,2% had long-term transfer over 4 months in the executive domain. In the memory domain 57,5% had short-term transfer, 57,5% showed maintenance of gains and 58% had long-term transfer. In the working memory domain, we found that 53,6% had short-term transfer and 63% of the participants showed long-term transfer. Here we observed that only 38,7% showed maintenance of gains, as opposed to 61,3% who did not maintain gains. The number of transferers and non-transferers in the CT and ACT + CT groups is listed in Supplementary Table 1.

### 3.3 Baseline cognitive performance and transfer effects of training

**Fig. 3:**
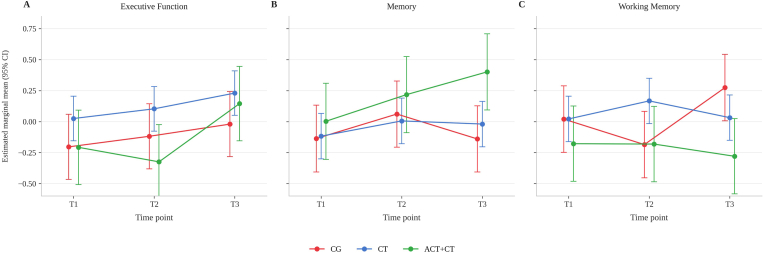
Mean aggregate scores with 95% CI. No significant group by time interaction was detected.

Our results demonstrated that the CT and ACT + CT groups did not differ significantly in the distribution of the scores on the baseline neuropsychological performance in executive functions *D* (118, 57) = 0.100, *p* = .785, memory *D* (118, 57) = 0.176, *p* = .159 and working memory *D* (118, 57) = 0.072, *p* = 978. Therefore, we included both groups in our analysis. When we examined the transfer effects of cognitive training, we did not find a significant group by time interaction (Fig. 3). Specifically, the group by time interaction was not significant on the executive functions scores (*F* (4, 462.01) = 0.77, *p* = .543, η^2^p = .007), indicating that the pattern of change over time did not differ significantly between the groups. The linear mixed model revealed a significant main effect of age (*F* (1, 226.04) = 15.53, *p* < .001, η^2^p = .06), indicating that older participants showed lower executive function scores across the three time points, with a moderate effect size. A significant main effect of time was also observed (*F* (2, 462.00) = 4.45, *p* = .012, η^2^p = .02), reflecting a general improvement in executive function scores across all groups over time. Given that this effect was present in all groups including the passive CG, it most likely reflects retest effects from repeated neuropsychological assessments rather than genuine training-induced improvement. Similarly, there was no significant group by time interaction on memory (*F* (4, 459.21) = 0.91, *p* = .457, η^2^p = .008). The same model revealed a significant main effect of sex (*F* (1, 224.54) = 9.85, *p* = .002, η^2^p = .04), indicating that male participants scored higher on the memory tasks across time. Furthermore, the group by time interaction was not significant on the working memory scores (*F* (4, 462.00) = 1.97, *p* = .098, η^2^p = .02).

### 3.4 Resting-state networks connectivity and association with transfer of training gains

#### 3.4.1 Seed-based connectivity analysis

The continuous linear regression analyses showed that baseline gFC did not significantly predict cognitive change in any of the three domains (all |β| ≤ .10, all p ≥ .097, all p-FDR ≥0.94). One of the logistic regression models indicated that high gFC of left DLPFC decreased the likelihood of having successful long-term transfer on the working memory domain (OR = 0.62, 95% CI [0.42,0.87], *p* < .05). Other logistic regression models did not demonstrate any significant association of gFC on the successful transfer or maintenance of training gains. In some models, sex was found to be a significant predictor of maintaining memory gains, indicating that women were less likely to maintain gains than men. In addition, some models indicated that a high baseline performance decreased the relative likelihood of short-term and long-term transfer of training gains in memory and working memory domains. The sensitivity analyses only with the CT group for both the linear and logistic regression models did not demonstrate significant results (Supplementary Table 2a and 2b).

#### 3.4.2 Independent component analysis

**Fig. 4:**
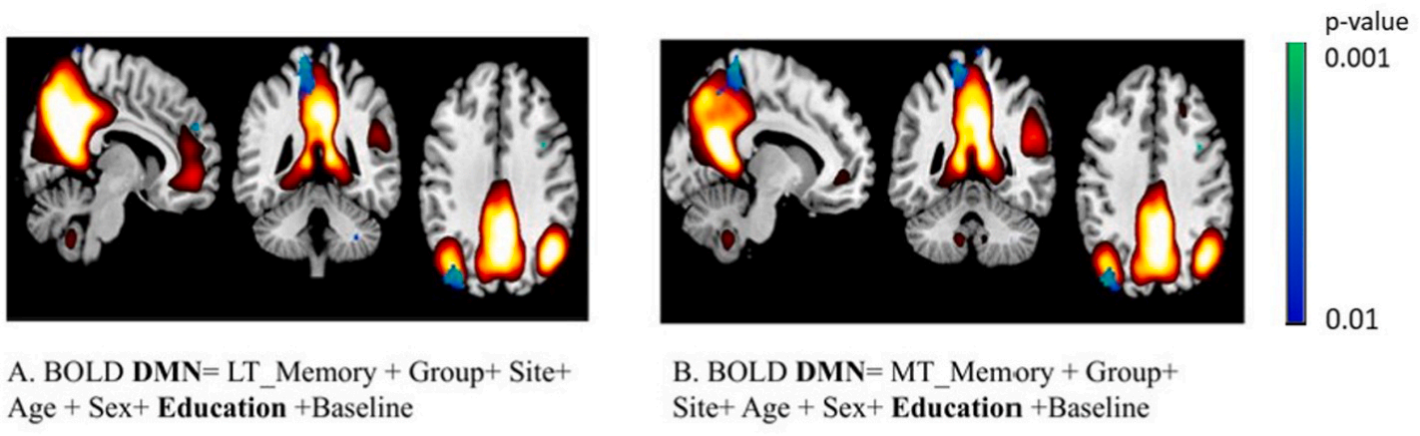
Results of the dual regression analysis of the resting-state fMRI. Yellow-white voxels represent DMN, blue voxels show significant association between network connectivity and education years on DMN (A, B) at p < .01 uncorrected. Association was restricted to the corresponding networks only by network-specific masks obtained from the independent component maps from the whole sample thresholded at p <. 05 TFCE, one-sample *t*-test. BOLD = blood oxygenation level-dependent, LT = long-term transfer, MT = maintenance of training gains.

Spatial maps derived from the whole-sample ICA decomposition of resting-state fMRI data corresponded to the DMN and the CEN. Results from the dual-regression did not demonstrate effects of baseline resting-state connectivity of the DMN and CEN on successful short- and long-term transfer, and maintenance of training gains in memory, working memory and executive function domains, at p < .01, uncorrected. Years of education had an effect on resting-state FC of DMN at p < .01 uncorrected (Fig. 4).

## 4 Discussion

We investigated the association between functional brain mechanisms and the transfer of cognitive training gains in healthy older adults.

First, we investigated whether near transfer of cognitive training gains was possible in a large sample of healthy older adults. We classified the participants in transferers and no transferers based on their improvement compared to the control group. Most of the participants had successful short-term and long-term transfer on executive function, memory and working memory domains. In addition, most of the participants maintained training gains on executive and memory domain. For working memory, the number of participants who maintained training gains was lower than the number of participants who did not maintain gains. This could be explained by the time point we defined transfer, which was the period between the post-training and follow-up phases. Our participants underwent a full baseline neuropsychological assessment with a total duration of ∼4 h and ∼3 h for the following two assessments. This means that they might have experienced cognitive overload after the baseline assessment and the cognitive training, which may have affected their post-training performance and subsequently the transfer measurements.

We should note that the definition of transfer in the current study was slightly different from that in the protocol paper (Wolf et al., 2018) and we observed that our results differed from those of our previous study (Faraza et al., 2021). In that study we analyzed an interim data set and demonstrated that transfer is limited in aging. The explanation for this discrepancy may be that in the current study we had a larger sample size, and we used aggregate scores. In addition, we had applied a stricter definition of long-term transfer in our previous study.

Considering that the majority of the participants had successful transfer, our results support previous findings, indicating that transfer effects occur in aging and that training gains can be maintained in the long-term (Basak et al., 2020; Borella et al., 2010; da Silva et al., 2022; Kelly et al., 2014; Noack et al., 2014; Zinke et al., 2014). Moreover, our findings are consistent with the neural overlap hypothesis, which suggests that transfer can be expected when the trained and transfer tasks share a common neurological component (Dahlin et al., 2008b; Lustig et al., 2009; Noack et al., 2014).

It is worth noting that the linear mixed-effects models did not reveal a significant group by time interaction, indicating that the training groups did not improve significantly more than the control group at the group level. The relatively high transfer rates therefore reflect individual-level improvement relative to the control group mean retest change, rather than a statistically robust group-level training effect. This distinction is important for interpreting the clinical significance of the transfer rates observed.

The primary continuous linear regression analyses showed that baseline resting-state FC did not significantly predict cognitive change in any domain, providing the most direct and statistically sensitive test of our primary research question. The secondary binary classification analyses conducted to ensure consistency with the pre-registered study protocol (Wolf et al., 2018) produced convergent null findings across all seed regions and cognitive domains. The null findings were also consistent across the CT-only sensitivity analyses for both approaches.

Furthermore, one of the logistic regression models showed that a high FC of the left dlPFC decreased the likelihood of a successful long-term transfer on working memory domain. This is consistent with the compensation-related utilization of neural circuits (CRUNCH) theory (Reuter-Lorenz and Cappell, 2008), which suggests that older adults over-recruit prefrontal areas to compensate for declining neural efficiency or age-related cognitive decline. However, contrary to our hypothesis, the rest of the logistic regression models showed no significant association between baseline FC of resting-state networks and successful transfer of cognitive training gains. One explanation could be the approach we used to investigate the FC of resting-state networks. We used *a priori* selected seed regions of the DMN and CEN. Our results showed no significant association between high global FC of *a priori* regions and the likelihood of successful transfer of training gains as well. The robustness of this null finding is consistent with the primary continuous linear regression analyses, in which baseline FC failed to predict cognitive change scores. Furthermore, ICA is a data-driven approach, which may not be sensitive enough in contrast to a seed-based approach.

A possible explanation for the lack of an association could be potential compensatory effects. As reported in the demographics section, our participants were highly educated. Previous studies have shown that education is a protective factor against age-related cognitive decline Clouston et al. (2020); Mondini et al. (2022); Arenaza-Urquijo et al. (2013) showed that higher education was associated with increased FC at rest in healthy older adults, which in turn was associated with improved cognitive performance. This is consistent with our findings, where we found effects of education on DMN connectivity. Montemurro et al. (2023) suggested that higher education contributes to the differentiation of neural patterns in brain at rest in healthy older adults. Specifically, when comparing groups of older adults with higher (12 years) and lower (10 years) education, they found an increased connectivity strength in the somatomotor network (SMN) and decreased connectivity strength in the DMN and visual-medial network (VMN) in adults with higher education. This could indicate the potential compensatory effects of these networks (Montemurro et al., 2023). A compensatory role of DMN areas has been reported also by Sala-Llonch et al. (2012). Our findings agree with the Scaffolding Theory of Aging and Cognition (Reuter-Lorenz and Park, 2014) which suggests that a high level of education is a protective factor that enables neurocognitive scaffolding. This means that cognitive function can be maintained despite neural deterioration. This may explain why the successful transfer of gains was not associated with FC of resting-state networks. The participants may have an age-related neural decline, but due to the protective factor of high education they maintained a high cognitive level. Furthermore, older adults compensate by selection, meaning that they recruit different brain regions than younger adults to compensate for decline (Cabeza et al., 2018). However, because we examined specific brain regions in this study, we could not test for this as a possible explanation. Our results may be interpreted within the cognitive reserve concept (Stern, 2009). We assume that our participants were cognitively agile, given that they were highly educated and were attracted to participate in a cognitive training study with long neuropsychological assessments. It is possible that they used cognitive resources in daily life and this is reflected in high cognitive performance.

In addition, we examined the role of demographic characteristics on the likelihood of a successful transfer. We built separate regression models for each transfer type and cognitive domain. Our findings indicated that sex was a predictor of transfer of training gains, suggesting that male participants were more likely to maintain training gains in the memory domain. Our results are consistent with the meta-analysis by Blume et al. (2010), which showed a small correlation between training transfer and male sex (ρ = 0.12, 80% CV [−0.12,0.37]). However, our results contrast previous research (Roheger et al., 2020) indicating that female sex was a predictor of gains (*β* = 0.26) after six weeks of training, but not after three months. Although individual differences such as age or baseline performance have been examined in the context of training gains in previous studies (Roheger et al., 2020; Zinke et al., 2014), evidence on sex differences is sparse and further research is needed. Regression models indicated that participants with high baseline performance on memory and working memory tests were less likely to have short-term and long-term transfer. Our results confirm previous findings (Roheger et al., 2020; Shaw and Hosseini, 2021; Zinke et al., 2014) suggesting that low baseline performance is associated with training success. This may reflect the definition of a successful transfer of training gains, which is defined as an improvement in the performance over baseline that is greater than the improvement in the control group. It is therefore possible that our participants reached the peak in their performance on the baseline assessment, and that further improvement was not possible.

Overall, our results provide evidence that there is no positive association between cognitive training gains and FC. A sample size of 235 healthy older adults in total and 175 older adults to examine our primary aim provides a statistical power of 0.8 (Wolf et al., 2018). This allows us to be confident that we are avoiding a Type II error and to support the negativity of the results, such that we can state that high FC is not a predictor of successful transfer of training gains in healthy older adults.

Our study has several limitations. The first limitation concerns the fMRI scan length. The resting-state fMRI scans in this study had a duration of 11.02 min with a TR of 1000 ms, and 10 vol were removed during preprocessing. A relatively short scan durations can produce noisy estimates of FC, potentially limiting sensitivity to detect brain-behavior associations (Birn et al., 2013).This might be an important factor in the explanation of the null results. Future studies should consider acquiring longer resting-state fMRI scans to improve the reliability of connectivity estimates. Moreover, GSR was selected as a denoising method. Although it is widely applied in fMRI studies, its use still remains controversial because it may eliminate relevant information to FC, such as age-related differences (Golestani and Chen, 2024; Liu et al., 2017) and may introduce negative correlation (J. Li et al., 2019). Future replication with alternative denoising approaches such as aCompCor or ICA-AROMA is recommended. Furthermore, the inclusion of a passive control group might not have been the ideal approach to examine transfer of gains. Future studies should include active and passive control groups. In addition, we defined successful transfer based on the performance at the individual level. Future studies could apply a different threshold to define successful transfer, which may increase statistical power. A further limitation concerns the operationalisation of maintenance of training gains. The maintenance classification required improvement from post-training to follow-up assessment that exceeded the mean change of the control group over the same period but did not require participants to have demonstrated short-term transfer at post-training. Consequently, some individuals were classified as maintainers without having shown prior improvement relative to the control group, which limits the interpretability of the maintenance of gains at the individual level. Future studies should consider requiring demonstrated short-term transfer as a prerequisite for maintenance classification, to ensure that the maintenance group genuinely reflects persistence of training-related gains. Another limitation concerns the two different approaches we followed to investigate FC. A seed-based approach has the advantage of *a priori* selection of seed-regions of interest. However, we did not examine FC of other brain regions. The ICA approach has the limitation of being a data-driven approach that generates FC networks from the whole sample. The resulting components were visually inspected and identified as networks based on previous literature and templates (Rosazza and Minati, 2011; Shirer et al., 2012). This means that the selected regions of networks may differ slightly from other studies. Another limitation is that the study design did not include post-training fMRI measurements, and this limited the possibility of observation of longitudinal functional changes and transfer effects.

## 5 Conclusions

The present study showed that transfer of cognitive training gains over a 4 month-period was possible in healthy aging. We did not find positive associations of training gains with FC in a large sample using both a *priori* and a data-driven method to investigate FC. Our findings lead us to conclude that functional connectivity patterns do not serve as a predictor of transfer of training gains in healthy older adults. In addition, our study raises the hypothesis that compensation mechanisms on brain network level may influence the likelihood of training gains in healthy aging. Future studies could investigate different predictive factors to determine the likelihood of training gains and how training gains can be generalized to other aspects of daily life.

## Ethics statements

The studies involving humans were approved by Mainz: Ethics Commission of the Landesärztekammer Rheinland-Pfalz, Rostock: Ethics Commission of the Rostock University's Faculty of Medicine, Cologne: Ethics Commission of (1) Cologne University's Faculty of Medicine and (2) German Sport University. Reference number of the ethics approval of the main trial site Mainz is 837.385.15 (10153). The studies were conducted in accordance with the local legislation and institutional requirements. The participants provided their written informed consent to participate in this study.

## Declaration of generative AI and AI-assisted technologies in the manuscript preparation process

During the preparation of the revised manuscript, the author(s) used Claude (Anthropic) in order to assist with language editing and restructuring of text. After using these tools, the author(s) reviewed and edited the content as needed and take(s) full responsibility for the content of the published article.

## Funding

This study was supported by a grant of the 10.13039/501100002347German Federal Ministry of Education and Research (10.13039/501100002347BMBF) (AgeGain, 01GQ1425B). The funding body has no influence on the study whatsoever.

## CRediT authorship contribution statement

**Sofia Faraza:** Formal analysis, Investigation, Methodology, Visualization, Writing – original draft. **Martin Dyrba:** Writing – review & editing. **Dominik Wolf:** Conceptualization, Funding acquisition, Investigation, Methodology, Project administration, Supervision. **Florian U. Fischer:** Conceptualization, Investigation, Methodology. **Kristel Knaepen:** Conceptualization, Investigation, Methodology. **David Riedel:** Conceptualization, Investigation. **Bianca Kollmann:** Conceptualization, Investigation, Methodology. **Harald Binder:** Conceptualization, Methodology, Project administration. **Andreas Mierau:** Conceptualization, Methodology, Project administration, Supervision. **Andreas Fellgiebel:** Conceptualization, Methodology, Project administration, Supervision. **Oliver Tüscher:** Conceptualization, Methodology, Project administration, Supervision. **Stefan Teipel:** Conceptualization, Methodology, Project administration, Supervision, Writing – review & editing.

## Declaration of competing interest

The authors declare that they have no known competing financial interests or personal relationships that could have appeared to influence the work reported in this paper.

## Data Availability

Data will be made available on request.
