# Supplementary material for: Resting state brain activity and association with transfer of cognitive training gains
Source: Neuroimage Rep. 2026 Jul 24;6(3):100389. doi: 10.1016/j.ynirp.2026.100389 (PMC13449371; doi:10.1016/j.ynirp.2026.100389)
Supplement: Supplementary file 1 [file mmc1.docx]

**Supplementary Material**

**AgeGain- Cognitive training tasks**

**TATOOL** ( Bastian et al. 2013)

**Complex Span**: Each trial started with a memory item (two-digit number). This was followed immediately by a distractor (number with one digit). Participants had to judge the parity (odd or even) of the digit. Afterward, the next memory item followed. After a few memory-decision sequences, participants had to recall the memoranda in the correct serial order. The number of memory items increased with the level of difficulty. Storage and processing of information were trained with these tasks.

**Tower of Fame:** Participants had to imagine a tower with apartments on several floors. Sentences were presented sequentially, giving information about which famous person lives in which apartment. At the end, participants had to recall which famous person lives where. Integration and coordination of information were trained with these tasks.

**COGPACK** (Marker Software)

**Anagrams:** Participants had to rearrange the letters of a meaningless word and create a meaningful word.

**Logic:** Traffic signs appeared and a text describing a condition. Participants had to click on the sign, which refers to the condition.

**Searching:** Letters appeared on the screen, only 1 letter appeared 3 times. Participants had to click on this letter.

**Remembering:** Series of words appeared (every word for 5 sec.). Participants had to memorize them and type them.

**Complete a Logical Block:** A series of signs appeared on the screen. Participants had to recognize the rule of the appearance order and continue the series following the rule.

**TAP Battery** (Zimmermann and Fimm, PsyTest)

**Alertness:** Reaction time was examined under two conditions. In the first condition a cross appears on the monitor at randomly varying intervals and to which the subject should respond as quickly as possible by pressing a key. Intrinsic alertness is measured in this condition. In the second condition, reaction time was measured in response to a critical stimulus preceded by a cue stimulus presented as warning tone ("phasic arousal", or temporal orientation of attentional focus).

**Divided attention**: A visual and an auditory task were processed in parallel.

Publication bibliography

Bastian, Claudia C. von; Locher, André; Ruflin, Michael (2013): Tatool: a Java-based open-source programming framework for psychological studies. In *Behavior research methods* 45 (1), pp. 108–115. DOI: 10.3758/s13428-012-0224-y.
