# Supplementary material for: Resting state brain activity and association with transfer of cognitive training gains
Source: Neuroimage Rep. 2026 Jul 24;6(3):100389. doi: 10.1016/j.ynirp.2026.100389 (PMC13449371; doi:10.1016/j.ynirp.2026.100389)
Supplement: Supplementary file 2 [file mmc2.docx]

**Supplementary Table 1.** Transferers and non-transferers across two groups

|  | CT ACT+CT | |
| --- | --- | --- |
|  | *n* | *n* |
| **Executive functions** |  |  |
| ST+/ST- | 73/51 | 32/25 |
| TM+/TM- | 76/48 | 35/22 |
| LT+/LT- | 103/21 | 44/13 |
| **Memory** |  |  |
| ST+/ST- | 69/55 | 35/22 |
| TM+/TM- | 68/56 | 36/21 |
| LT+/LT- | 68/56 | 37/20 |
| **Working memory** |  |  |
| ST+/ST- | 67/57 | 30/27 |
| TM+/TM- | 48/76 | 22/35 |
| LT+/LT- | 79/45 | 35/22 |

_______________________________________

*Note*. *CT*= Cognitive Training Group,
*CT+ACT=* Cognitive Training + Aerobic Coordination Training Group
