# Supplementary material for: Resting state brain activity and association with transfer of cognitive training gains
Source: Neuroimage Rep. 2026 Jul 24;6(3):100389. doi: 10.1016/j.ynirp.2026.100389 (PMC13449371; doi:10.1016/j.ynirp.2026.100389)
Supplement: Supplementary file 3 [file mmc3.docx]

**Supplementary Table 2a.** Linear regression results of CT-only sensitivity analyses

**Executive Function	Seed	B	SE	t	p	p-FDR**

_______________________________________________________________________

Short-term	PCC	0.082	0.081	1.018	.311	.955

mPFC	0.049	0.081	0.607	.545	.955

lPC	0.015	0.080	0.183	.855	.955

rdlPFC	-0.067	0.080	-0.845	.400	.955

ldlPFC	-0.006	0.078	-0.079	.937	.955

ACC	0.072	0.080	0.911	.364	.955

Long-term	PCC	-0.060	0.081	-0.738	.462	.955

mPFC	0.114	0.081	1.397	.165	.955

lPC	-0.034	0.081	-0.417	.678	.955

rdlPFC	0.042	0.080	0.522	.603	.955

ldlPFC	0.009	0.079	0.118	.906	.955

ACC	0.089	0.080	1.118	.266	.955

Maintenance	PCC	-0.126	0.100	-1.263	.209	.955

mPFC	0.059	0.101	0.581	.562	.955

lPC	-0.043	0.100	-0.433	.666	.955

rdlPFC	0.097	0.099	0.980	.329	.955

ldlPFC	0.014	0.097	0.142	.887	.955

ACC	0.017	0.099	0.168	.867	.955

**Memory**

Short-term	PCC	-0.083	0.073	-1.136	.259	.955

mPFC	0.031	0.074	0.426	.671	.955

lPC	0.026	0.073	0.359	.720	.955

rdlPFC	0.072	0.072	1.001	.319	.955

**Memory	Seed	B	SE	t	p	p-FDR**

_______________________________________________________________________

ldlPFC	-0.064	0.070	-0.904	.368	.955

ACC	0.045	0.072	0.623	.534	.955

Long-term	PCC	0.029	0.071	0.416	.678	.955

mPFC	0.066	0.071	0.926	.356	.955

lPC	0.007	0.071	0.098	.922	.955

rdlPFC	-0.028	0.070	-0.397	.692	.955

ldlPFC	-0.078	0.068	-1.151	.252	.955

ACC	-0.029	0.070	-0.422	.674	.955

Maintenance	PCC	0.127	0.098	1.304	.195	.955

mPFC	0.039	0.099	0.395	.693	.955

lPC	-0.022	0.098	-0.223	.824	.955

rdlPFC	-0.113	0.096	-1.174	.243	.955

ldlPFC	-0.017	0.095	-0.175	.862	.955

ACC	-0.085	0.097	-0.873	.385	.955

**Working Memory**

Short-term	PCC	0.099	0.075	1.332	.186	.955

mPFC	0.039	0.075	0.522	.603	.955

lPC	-0.002	0.075	-0.025	.980	.980

rdlPFC	-0.055	0.073	-0.759	.449	.955

ldlPFC	-0.064	0.072	-0.882	.380	.955

ACC	-0.013	0.073	-0.178	.859	.955

Long-term	PCC	0.047	0.078	0.600	.550	.955

mPFC	-0.061	0.077	-0.787	.433	.955

lPC	0.022	0.077	0.283	.778	.955

rdlPFC	-0.012	0.076	-0.158	.875	.955

**Working Memory	Seed	B	SE	t	p	p-FDR**

_______________________________________________________________________

ldlPFC	-0.015	0.075	-0.200	.842	.955

ACC	0.045	0.076	0.601	.549	.955

Maintenance	PCC	-0.040	0.100	-0.400	.690	.955

mPFC	-0.102	0.100	-1.029	.306	.955

lPC	0.025	0.099	0.257	.798	.955

rdlPFC	0.038	0.097	0.386	.700	.955

ldlPFC	0.042	0.096	0.434	.665	.955

ACC	0.061	0.097	0.632	.529	.955

*Note:* B = unstandardised linear regression coefficient (z-standardised outcome and predictor); SE = standard error; t = t-statistic; p = two-tailed p-value; p-FDR = p-value after Benjamini-Hochberg false discovery rate correction.

**Supplementary Table 2b.** Logistic regression results of CT-only sensitivity analyses

**Executive Function	Seed	B	SE	OR	Z	p	p-FDR**

_____________________________________________________________________

Short-term transfer	PCC	-2.391	2.844	0.092	-0.841	.401	.999

mPFC	-0.772	3.859	0.462	-0.200	.842	.999

lPC	2.195	3.995	8.983	0.549	.583	.999

rdlPFC	-1.940	3.595	0.144	-0.540	.589	.999

ldlPFC	-1.604	3.357	0.201	-0.478	.633	.999

ACC	1.619	3.223	5.050	0.502	.615	.999

Long-term transfer	PCC	-2.992	3.370	0.050	-0.888	.375	.999

mPFC	-3.272	4.825	0.038	-0.678	.498	.999

lPC	1.130	5.084	3.095	0.222	.824	.999

rdlPFC	-5.719	4.205	0.003	-1.360	.174	.999

ldlPFC	-1.733	4.185	0.177	-0.414	.679	.999

ACC	-0.409	3.898	0.664	-0.105	.916	.999

Maintenance	PCC	-0.880	2.870	0.415	-0.306	.759	.999

mPFC	-1.686	3.888	0.185	-0.434	.664	.999

lPC	0.284	3.952	1.328	0.072	.943	.999

rdlPFC	-2.029	3.630	0.131	-0.559	.576	.999

ldlPFC	-0.263	3.348	0.769	-0.078	.937	.999

ACC	0.014	3.069	1.014	0.004	.997	.999

**Memory**

Short-term transfer	PCC	3.735	3.018	41.871	1.238	.216	.999

mPFC	4.746	3.923 115.167	1.210	.226	.999

lPC	4.579	3.989	97.426	1.148	.251	.999

rdlPFC	1.647	3.549	5.193	0.464	.642	.999

**Memory	Seed	B	SE	OR	Z	p	p-FDR**

_____________________________________________________________________

ldlPFC	1.587	3.342	4.889	0.475	.635	.999

ACC	-2.524	3.049	0.080	-0.828	.408	.999

Long-term transfer	PCC	-0.265	2.858	0.767	-0.093	.926	.999

mPFC	0.006	3.819	1.006	0.002	.999	.999

lPC	0.051	3.872	1.053	0.013	.989	.999

rdlPFC	-0.118	3.558	0.889	-0.033	.974	.999

ldlPFC	-3.661	3.334	0.026	-1.098	.272	.999

ACC	-1.642	3.070	0.194	-0.535	.593	.999

Maintenance	PCC	0.885	2.852	2.423	0.310	.756	.999

mPFC	-0.410	3.782	0.664	-0.108	.914	.999

lPC	-0.978	3.838	0.376	-0.255	.799	.999

rdlPFC	-0.656	3.533	0.519	-0.186	.853	.999

ldlPFC	0.858	3.339	2.357	0.257	.797	.999

ACC	-0.575	3.033	0.562	-0.190	.850	.999

**Working memory**

Short-term transfer	PCC	-2.031	3.155	0.131	-0.644	.520	.999

mPFC	-0.367	4.060	0.693	-0.090	.928	.999

lPC	-2.212	4.173	0.109	-0.530	.596	.999

rdlPFC	-1.675	3.800	0.187	-0.441	.659	.999

ldlPFC	-5.280	3.843	0.005	-1.374	.170	.999

ACC	-2.394	3.315	0.091	-0.722	.470	.999

Long-term transfer	PCC	0.948	3.063	2.581	0.310	.757	.999

mPFC	-0.198	4.143	0.821	-0.048	.962	.999

lPC	-7.068	4.362	0.001	-1.621	.105	.999

rdlPFC	-8.155	4.035	0.000	-2.021	.043	.999*

**Working memory	Seed	B	SE	OR	Z	p	p-FDR**

___________________________________________________________________________

ldlPFC	-12.911	4.453	0.000	-2.899	.004	.200*

ACC	-3.762	3.358	0.023	-1.120	.263	.999

Maintenance	PCC	1.079	2.894	2.943	0.373	.709	.999

mPFC	-4.727	4.089	0.009	-1.156	.248	.999

lPC	-0.619	3.978	0.538	-0.156	.876	.999

rdlPFC	-2.710	3.723	0.067	-0.728	.467	.999

ldlPFC	-3.743	3.643	0.024	-1.027	.304	.999

ACC	-1.169	3.219	0.311	-0.363	.717	.999

__________________________________________________________________________

*Note*: precuneus (PCC); medial prefrontal cortex (mPFC); lateral parietal cortex (lPC); right dorsolateral prefrontal cortex (rdlPFC); left dorsolateral prefrontal cortex (ldlPFC); anterior cingulate cortex (ACC); B = unstandardized logistic regression coefficient. SE = standard error; OR = odds ratio; Z = Wald z-statistic; p = two-tailed p-value; p-FDR = p-value after Benjamini-Hochberg false discovery rate correction.

* p < .05 (uncorrected); no effects survived FDR correction.
